# Monitoring Multiple Behaviors in Beef Calves Raised in Cow–Calf Contact Systems Using a Machine Learning Approach

**DOI:** 10.3390/ani14223278

**Published:** 2024-11-14

**Authors:** Seong-Jin Kim, Xue-Cheng Jin, Rajaraman Bharanidharan, Na-Yeon Kim

**Affiliations:** 1 Asia Pacific Ruminant Institute, Icheon 17385, Republic of Korea; apri76@daum.net (S.-J.K.); kimhs@live.com (X.-C.J.); 2 Department of Eco-Friendly Livestock Science, Institute of Green Bio Science and Technology, Seoul National University, Pyeongchang 25354, Republic of Korea; bharanidharan7@snu.ac.kr

**Keywords:** machine learning, multiple behaviors, natural suckling, coughing, beef calves, cow–calf contact systems, inertial measurement unit

## Abstract

The monitoring of pre-weaned calf behavior is crucial for ensuring health, welfare, and optimal growth. This study aimed to develop and validate a machine learning-based technique for the simultaneous monitoring of multiple behaviors in pre-weaned beef calves within a cow–calf contact (CCC) system using collar-mounted sensors integrating accelerometers and gyroscopes. Three complementary models were developed to classify feeding-related behaviors (natural suckling, feeding, rumination, and others), postural states (lying and standing), and coughing events. Sensor data, including tri-axial acceleration and tri-axial angular velocity, along with video recordings, were collected from 78 beef calves across two farms. The LightGBM algorithm was employed for behavior classification, and model performance was evaluated using a confusion matrix, the area under the receiver operating characteristic curve (AUC-ROC), and Pearson’s correlation coefficient (r). Model 1 achieved a high performance in recognizing natural suckling (accuracy: 99.10%; F1 score: 96.88%; AUC-ROC: 0.999; r: 0.997), rumination (accuracy: 97.36%; F1 score: 95.07%; AUC-ROC: 0.995; r: 0.990), and feeding (accuracy: 95.76%; F1 score: 91.89%; AUC-ROC: 0.990; r: 0.987). Model 2 exhibited an excellent classification of lying (accuracy: 97.98%; F1 score: 98.45%; AUC-ROC: 0.989; r: 0.982) and standing (accuracy: 97.98%; F1 score: 97.11%; AUC-ROC: 0.989; r: 0.983). Model 3 achieved a reasonable performance in recognizing coughing events (accuracy: 88.88%; F1 score: 78.61%; AUC-ROC: 0.942; r: 0.969). This study demonstrates the potential of machine learning and collar-mounted sensors for monitoring multiple behaviors in calves, providing a valuable tool for optimizing production management and early disease detection in the CCC system

## Simple Summary

Monitoring the behavior of young calves is essential for ensuring their health, welfare, and optimal growth. However, traditional observation methods are time-consuming and labor-intensive. This study aimed to develop a technology-based solution for automatically monitoring multiple behaviors in beef calves raised with their mothers. Collar-mounted sensors, which combined accelerometers and gyroscopes, were used to collect data on calf movements. Machine learning techniques were then applied to analyze the data and classify behaviors related to feeding, posture, and coughing. The results showed that the developed models could accurately identify these behaviors, providing a powerful tool that allows farmers to optimize their calf management strategies and detect potential health issues early on. This technology can help improve the efficiency and sustainability of calf rearing practices, ultimately benefiting both farm productivity and animal welfare.

## 1. Introduction

Monitoring the behavior of pre-weaned calves is crucial in farm management, as changes in their behavior often serve as early indicators of health issues, reflecting both their welfare and growth status. Bovine respiratory disease (BRD) and neonatal calf diarrhea (NCD) are the major causes of mortality in young calves [1]. These diseases are frequently accompanied by noticeable changes in calf behavior, particularly, feeding behavior, rumination, and lying behavior [2,3,4,5,6,7]. In particular, coughing is recognized as a classic clinical manifestation of BRD [8]. In addition, understanding rumination and solid feed intake patterns provides insights into rumen development and growth performance, assisting farmers in determining optimal weaning times [9,10]. Therefore, monitoring these behaviors contributes to maintaining the health and welfare of the calves and provides valuable information for optimizing their growth conditions.

The traditional observation of calf behavior requires substantial labor and time, which has encouraged the development and application of various precision livestock farming (PLF) technologies. Sensor devices, such as accelerometers, automatic calf feeding systems (ACFS), temperature boluses, and a range of cameras, have become the primary tools for the continuous, long-term collection of data about individual animals [11]. Among these devices, accelerometers stand out due to their non-invasive, broad monitoring capability and flexible mobility [12]. Nevertheless, the monitoring characteristics of accelerometers differ based on their placement on calves, such as on the limbs, neck, ears, or halter [13,14,15,16]. For instance, accelerometers attached to the legs can record the lying and standing behaviors of calves but are limited when measuring rumination and feeding behaviors [17]. Given this, several studies have attempted to integrate two or more sensors for the synchronized monitoring of multiple behaviors [5,18,19]. Considering the increased equipment and maintenance costs, this approach is not easily adopted in actual farming situations. Thus, the development of a sensor capable of accurately monitoring multiple behaviors simultaneously is a pressing focus of current research.

The cow–calf contact (CCC) system is defined as a rearing approach that allows physical contact between a cow and her own calf or between a foster cow and a foster calf, enabling behaviors such as licking, sniffing, suckling/nursing, and playing [20]. This system contrasts with traditional dairy farming practices, where newborn calves are quickly separated from their mothers to maximize milk yields. This leads them to primarily rely on manual feeding or automated methods like the use of ACFS [21]. In contrast, in beef cattle farming, whether in grazing or intensive confinement production systems, calves typically suckle directly from their mothers [22]. This natural suckling behavior promoted by the CCC system promotes positive cow–calf interactions, enhancing calf welfare and weight gain, while also increasing flexibility during the working days of farmers [23,24,25]. Due to these advantages, both consumers and dairy farmers are showing increasing interest in this CCC system [26]. However, this system also faces challenges. The milk intake of calves is closely linked to the nutritional status and body condition of the cow [27]. Unlike ACFS, which have sensors to record milk intake, determining the actual milk consumption in a CCC system is difficult. Additionally, there are concerns regarding real-time monitoring of sickness behavior in calves and the stress associated with transitioning to solid feeds during weaning [28,29]. These issues underscore the need for farmers using the CCC system to be informed of calf suckling and other related behaviors quickly, enabling intervention when necessary.

Machine learning algorithms are capable of modeling diverse and complex datasets to characterize animal behavior [30]. This approach has undergone extensive investigation in adult cattle [31,32,33]. In a recent study, Carslake et al. [34] utilized the AdaBoost ensemble learning algorithm and neck-worn collar sensors to classify multiple behaviors, including ruminating, suckling, and lying, in dairy calves raised in an ACFS. In contrast to ACFS, the CCC system allows calves to suckle naturally from their mothers in various positions and angles, making it more challenging to classify their behaviors, especially natural suckling. To the best of the authors’ knowledge, there is a lack of research on the classification of multiple behaviors, including natural suckling, in calves within the CCC system. Therefore, the current study aimed to employ machine learning in conjunction with accelerometers and gyroscopes to develop and validate a technique for simultaneous monitoring of multiple behaviors in pre-weaned beef calves in the CCC system, thereby enhancing productivity and ensuring high-quality animal welfare.

## 2. Materials and Methods

### 2.1. Animals and Housing

This study was conducted with the approval of the Animal Experimentation Ethics Committee (SNU-221228-2). Two beef cattle farms with different suckling systems were selected for two types of experiments. A total of 78 Korean native beef calves were included in this study, with 48 calves (19 male and 29 female) in Experiment 1 and 30 calves (11 male and 19 female) in Experiment 2.

Experiment 1 aimed to develop and validate monitoring models for natural suckling, feeding, ruminating, lying, and standing behaviors. The experiment was conducted from 19 November 2020 to 7 January 2022, at Farm A in Eumseong County, Chungcheongbuk-do, South Korea. Four Korean native cow–calf pairs were housed together in a pen measuring 32 m^2^ (8 m long × 4 m wide). Calves had free access to an adjoining calf pen (measuring 8 m^2^, 2 m long × 4 m wide). The calves naturally suckled colostrum from their dams within 3 h after birth and had free access to nurse throughout the pre-weaning period. Water was provided from birth, and commercial starter feed (Special baby, Farmsco Inc., Anseong, Republic of Korea) and oat hay were offered ad libitum when the calves were 3 days old. The chemical compositions of the feeds are presented in Table S1. Wood shavings were spread on the floor to a height of 10 cm and were regularly replaced to maintain a dry environment. At midday in summer, natural light penetration through the roof was adjusted to provide sufficient shade, and ventilation fans were operated adequately to prepare for heat stress. In winter, wind chill was prevented using winch curtains, and heat lamps were installed in the areas the calves were allowed to go. The calves were weaned from their dams at 90 days of age. The average birth weight of the calves was 26.1 ± 4.3 kg, and the weaning weight was 91.7 ± 12.0 kg.

Experiment 2 was conducted from 4 November 2020 to 31 March 2022, at Farm B located in Icheon-si, Gyeonggi-do, Republic of Korea, to develop and validate the cough monitoring model. All calves were separated from their dams immediately after birth and housed individually in cages measuring 2.5 m^2^ (2 m long × 1.25 m wide). Calves were fed 600 to 900 g of commercial dried colostrum (Harmony, Cheonhajeil Feed Inc, Daejeon, Republic of Korea) within 4 h of birth. From day 1 to day 15, calves were manually fed milk replacer (MR) at a concentration of 150 g/L twice daily (08:00 h and 17:00 h) with a maximum intake of 4 L per day. From day 3 onwards, water, starter feed (identical to Experiment 1, Farmsco), and timothy hay (5% of total solid feed, as-fed basis) were provided ad libitum. From day 16 to day 30, MR was offered at a maximum of 6 L daily, divided into five feedings through an automated milk feeding system (Calfrail, Forster, Engen, Germany). The amount of MR was linearly decreased from day 31 until the supply was terminated at day 60. The proportion of timothy hay in the total solid diet was increased to 15% (as-fed basis) on day 40. All calves were weaned at 60 days of age, as they consistently consumed over 1 kg of solid feed per day by this time point. Following a 7-day weaning adaptation period, calves were moved to group housing pens (area: 100 m² = 10 m long × 10 m wide, accommodating up to 13 calves) and remained there until 90 days of age. After moving, the calves had ad libitum access to high-quality concentrated feed, timothy hay, and water. The feed and water were replaced daily, and the chemical compositions of the feeds are presented in Table S1. Wood shavings were spread on the floor to a height of at least 10 cm and were regularly replaced to maintain a dry environment. In summer, natural light penetration through the roof was adjusted to provide sufficient shade, and ventilation fans and misting systems were operated adequately to prepare for heat stress. In winter, wind chill was prevented using winch curtains, and heat lamps were installed for each individual cage. The average birth weight of the calves was 30.5 ± 4.2 kg, and the weaning weight was 70.2 ± 9.9 kg. The monthly average, maximum, and minimum temperatures of the farms during the experimental period are shown in Figure S1.

### 2.2. Behavioral Data Collection

At Farm A, samples of natural suckling, feeding, rumination, lying, and standing behaviors of calves under the CCC system were collected. Farm B, which utilized individual cages or group calf pens, had a layout that minimized blind spots compared to Farm A. This arrangement facilitated easier visual observation of subtle movements.

**Figure 1:**
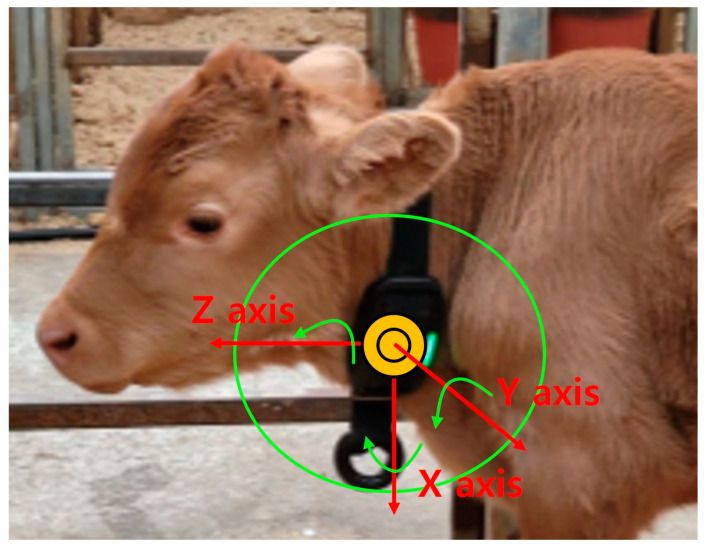
Schematic of the wearing position of the sensor device. The six axes of the IMU (integrating accelerometers and gyroscopes) are displayed.

Inertial measurement unit (IMU) sensors and internet protocol (IP) cameras were utilized to obtain experimental and observational data from the calves. The sensor device (FH Sensor, Bodit Inc., Seoul, Republic of Korea) integrated a six-axis IMU sensor consisting of an accelerometer and gyroscopes. This device measured 130 mm × 67 mm × 33 mm (L × W × H) and weighed 160 g, making it suitable for use with calves. Signals were collected for all six axes at a frequency of 25 Hz with the scales set at 4 *g* for acceleration and 500 degrees per second for angular velocity. The collected data were transmitted to a gateway via Bluetooth low energy (BLE) 4.2 technology. The sensor devices were worn as collars on the left sides of the calves’ necks for data collection (Figure 1). The same sensor devices and data collection methods were employed on both farms.

**Figure 2:**
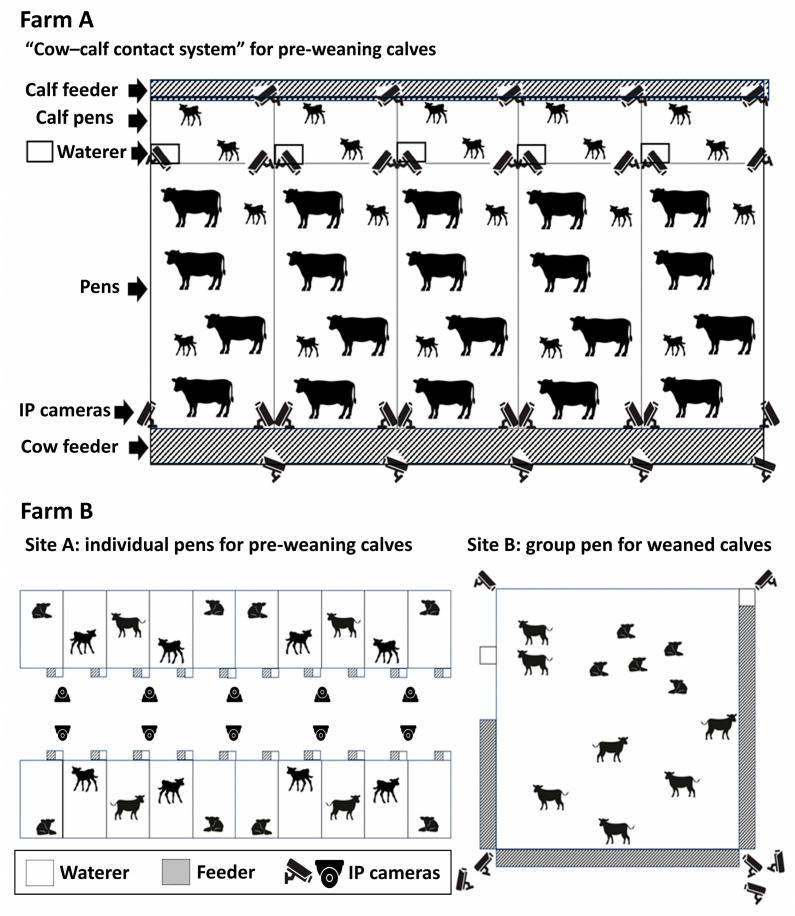
Schematic diagram of the calf houses’ structures and IP camera placement at Farms A and B.

Video data were collected using 30 IP cameras at Farm A and 17 IP cameras at Farm B. Due to the small sizes of the calves and potential obstruction by the dam cows, the cameras at Farm A were installed in groups of six per direction in five total directions to minimize blind spots. At Farm B, two individual cages were displayed closely in the view of one camera (Site A) to observe actions performed within a small range, such as the coughing of pre-weaned calves. For the group pens with weaned calves (Site B), seven cameras were strategically placed at various angles to minimize blind spots (Figure 2).

### 2.3. Behavior Tag Processing

**Figure 3:**
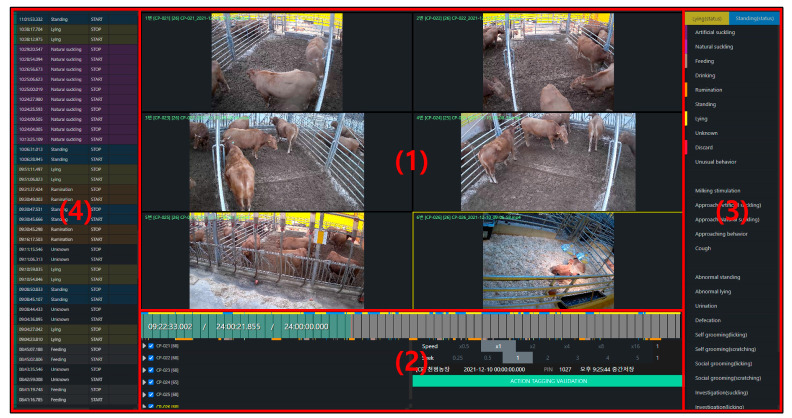
Interface of a typical animal behavior tagging software. (1) IP camera playback screen allowing 24-h video review and simultaneous viewing of six channels from various angles. (2) Playback control bar with options for quick time navigation, speed adjustment, and interval settings. (3) Behavior tagging buttons for recording the onset and conclusion of specific behaviors. (4) Tagging record display.

Twelve observers monitored the calves’ 24 h movements using observational data collected from IP cameras. All observers were trained to tag behaviors and used the animal behavior video annotation program (BoditToolBox, version 1.0, Bodit Inc.) to directly observe and tag the calves, as shown in Figure 3. Each observer performed this task for a period of six months. Descriptions of each type of collected behavior are presented in Table 1.

To minimize observation errors and establish reliability, pairs of two observers observed the same calf daily for 10 days. Observations were conducted in separate spaces to minimize interference and ensure objectivity. Data from all observers were used. Specifically, two pairs observed 10 calves each, while the remaining pairs observed nine calves each. In total, 112 observations were collected over the 10 days, and Cohen’s kappa coefficient was calculated for comparison [35]. The formula was as follows:

(1) $$K=\frac{P_{0}-P_{c}}{1-P_{c}}$$

where $P_{0}$ is the proportion of observed agreements and $P_{c}$ is the proportion of agreements expected by chance. As a result, the Cohen’s kappa coefficient reached a reliable level with an average of 94.60 ± 0.03. Subsequently, all observers individually observed different calves.

Through all tagging operations, ground truth data were obtained for the calves. The numbers of days with true values were 854 for Experiment 1 and 123 for Experiment 2. Dates with power outages affecting sensor data, dates when sensor collars were detached, and dates with sensor malfunctions were excluded from the true values.

### 2.4. Behavioral Classification Models

To classify calf behavior simultaneously, the model was divided into three parts. In Model 1, four behaviors were classified: natural suckling, rumination, feeding, and other behaviors. These behaviors involve prolonged and continuous movements over extended periods. Any behavior not categorized as natural suckling, rumination, or feeding was grouped as “others” in the miscellaneous category.

In Model 2, calf behavior was classified into two categories: lying and standing. Separation of Model 1 and Model 2 was necessary because the behaviors identified in these models can occur simultaneously. For example, a calf may engage in natural suckling while standing and exhibit rumination behavior while lying down.

Model 3 was designed to independently predict coughing events at specific points in time. This separation allowed the model to focus on discerning brief movements independent of the more extended and continuous behaviors addressed by Models 1 and 2.

### 2.5. Preprocessing and Labeling

To extract features, the original signals of acceleration and angular velocity as well as the magnitudes of acceleration and angular velocity were computed [36]. The formulas were as follows:

(2) $$A_{m}=\sqrt{{a_{x}}^{2}+{a_{y}}^{2}+{a_{z}}^{2}}$$

(3) $$G_{m}=\sqrt{{g_{x}}^{2}+{g_{y}}^{2}+{g_{z}}^{2}}$$

Here, $a_{x}$, $a_{y}$, and $a_{z}$ are the signals along the *x*, *y*, and *z* axes of the accelerometer, and $g_{x}$, $g_{y}$, and $g_{z}$ are the signals along the *x*, *y*, and *z* axes of the gyroscope. *A_m_* represents the magnitude of the acceleration, and *G_m_* represents the magnitude of the angular velocity.

Different signal preprocessing methods were applied depending on the model. First, for Models 1 and 2, time series data were converted into window units with a size of 10 s and 50% overlap. Each window contains 250 samples of data from the time series. The class of each window was assigned based on the action that occupied more than 50% of the window. For example, if rumination occurred for more than 5 s in a lying position, the window was labeled with the action class “rumination” and the state class “lying”. If the action occurred for less than 50% of the window, it was classified in the “others” category.

For Model 3, as coughing behavior involves short, subtle movements that last less than 2 s and occur infrequently throughout the day, converting all time series into short windows was computationally expensive and inefficient. Instead, a different approach was taken. The acceleration magnitude ($A_{m}$) was used to perform peak detection, extracting local maximum values above a specific threshold within a particular time range [37]. The extracted positions, referred to as peak points, and their corresponding values, peak values, were obtained. Using the extracted peak points as references, windows were created for 31 samples of time series data, including the 15 samples that preceded and followed the peaks. If a cough was tagged within a window, it was labeled as the coughing class; otherwise, it was labeled as the non-coughing class.

**Figure 4:**
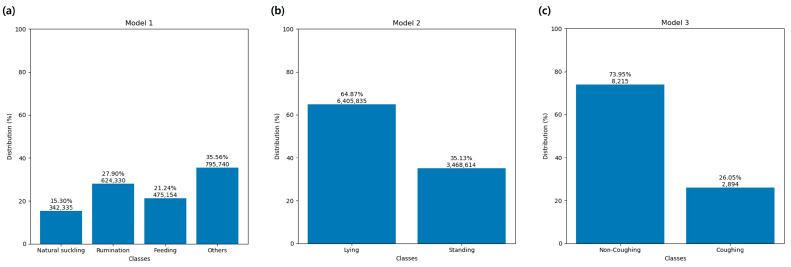
Class distributions of (**a**) Model 1 (natural suckling, rumination, feeding, and others), (**b**) Model 2 (lying and standing), and (**c**) Model 3 (coughing and non-coughing).

Figure 4 shows the distributions of the labeled classes for each model. In Model 3, there was an imbalance between the coughing and non-coughing classes due to a lack of coughing data when the calves did not have any respiratory issues.

### 2.6. Feature Extraction and Selection

Features were extracted not only from the time domain but also from the frequency domain. Although various types of features extracted from acceleration signals have been suggested [36,38], meaningful features need to be extracted or selected before modeling for a machine learning model to demonstrate optimal performance. In this experiment, the goal was to observe behaviors from a domain perspective and extract types of features that effectively represent the characteristics of each behavior. The desired features for each behavior are outlined below.

For natural suckling, a characteristic was observed when the calf moved its head up and down while nursing. To represent this feature in the signal, the movement of the rotation axis (angular velocity Y) in the nodding direction was investigated. A discrete Fourier transform was utilized to identify periodicity, as shown below [39].

(4) $$X\left[k\right]=\sum_{n=0}^{N-1}x\left[n\right]\cdot e^{-j\frac{2\pi }{N}kn}\;k=0,\;1,\;2,\;\ldots ,\;N-1$$

where

- *X*[*k*] represents the complex representation of the signal in the frequency domain.
- *x*[*n*] is the *n*-th sample of the accelerometer and gyroscope signals.
- *N* is the length of the time-domain signal, representing the total number of frequency points in the DFT.
- *j* is an imaginary unit.

The calculated frequency signal was enhanced by adding the frequency with the highest power and its corresponding power value.

For rumination and feed intake, where the sensor’s axis direction generally aligned when lying or lowering the head, additional features were introduced. Both behaviors include repetitive chewing movements, so the interquartile range (*IQR*) and the number of zero crossings for the axis were included.

(5) IQR=Q3−Q1

where

- *Q*1 is the first quartile, representing the lower 25% of the data.
- *Q*3 is the third quartile, representing the upper 75% of the data.

For lying and standing behaviors, where movement intensity varies, the variance and standard deviation of the signal were extracted to gauge its stability and variability.

For coughing, a feature was added to capture short and rapid movements of the sensor utilizing the gradient of the signal.

(6) $$Gradient\;G\left[n\right]=\frac{X\left[n+1\right]-X\left[n\right]}{∆t}\;n=0,\;1,\;2,\;\ldots ,\;N-1$$

where

- $G\left[t\right]$ is the *n*-th gradient of the signal.
- $x\left[n+1\right]$ is the (n+1)-th sample of the signal.
- $x\left[n\right]$ is the n-th sample of the signal.
- N is the length of the signal.
- ∆t is the time interval between the previous sample and the current sample.

To refine the set of features, highly correlated features were removed, and features with low importance according to a decision tree-based analysis were eliminated. The tree-based feature importance analysis was conducted using feature importance scores obtained from a decision tree model. This model evaluates how important each feature is during the process of building the tree structure. This helps to easily identify features that have a significant impact on the predictive performance of the model. Consequently, Model 1 had a total of 250 extracted features; Model 2 had 101 features; and Model 3, which aimed to identify coughing, had a total of 76 features.

### 2.7. Algorithm and Validation

The behavior of a calf was predicted using LightGBM, a gradient-boosting framework based on the decision tree algorithm [40]. This is a supervised machine learning algorithm that uses a leaf-wise strategy to build and split trees.

For Models 1 and 2, the training and testing datasets were divided based on individual calves. To mitigate the risk of overfitting, 42 calf data were randomly allocated for training and six calf data were randomly allocated for testing. This approach ensured that certain individual data did not dominate when all data were randomly split and that the model’s accuracy was robust when applied to new individuals. Furthermore, within the training dataset, an additional random split was performed at an 8:2 ratio to create training and validation datasets for model tuning and validation.

For Model 3, the training and validation datasets were pre-divided randomly at an 8:2 ratio. Due to its smaller dataset size compared to the other models, five-fold cross-validation was applied. Additionally, since the coughing data exhibited an imbalanced distribution with a 2.8:1 ratio, the imbalance issue was addressed by oversampling the minority class (coughing) in the training dataset. Oversampling was achieved using the SMOTE technique [41].

All hyperparameter tuning for the models was conducted using the validation dataset. The Optuna hyperparameter optimization framework was utilized to automatically find the optimal model parameters (including learning rate, number of estimators, and max depth) through iterative evaluations [42].

### 2.8. Evaluation

To evaluate the models, the following metrics were considered: accuracy, true-positive rate (*TPR*), true-negative rate (*TNR*), positive-predictive value (*PPV*) and F1 score. Additionally, a confusion matrix was examined to visualize the performance of the multiclass classification model. The formulas for the evaluation metrics were as follows:

(7) $$Accuracy=\frac{TP+TN}{TP+TN+FP+FN}$$

(8) $$TPR=\frac{TP}{TP+FN}$$

(9) $$TNR=\frac{TN}{TN+FP}$$

(10) $$PPV=\frac{TP}{TP+FP}$$

(11) $$F1\;score=2\times \frac{PPV\times TPR}{PPV+TPR}$$

Here, *TP* represents true positives, *TN* represents true negatives, *FP* represents false positives, and *FN* represents false negatives.

Furthermore, for Models 1 to 3, receiver operating characteristic (ROC) curves were plotted, and the area under the curve (AUC) was calculated at all classification thresholds to assess the model’s performance [43]. The ROC curve displayed the TPR and the false-positive rate (*FPR*), which was defined as follows:

(12) $$FPR=\frac{FP}{TN+FP}$$

To validate the models, Pearson’s correlation coefficient (r) was computed to compare the sensor’s predicted results with the actual observational outcomes. The predictions were initially given as behavior classes, each corresponding to a 10 s window. These class predictions were converted into numerical values by assigning 10 s to each class, enabling the calculation of the correlation coefficient. For Models 1 and 2, the predicted time for each behavior was compared with the actual observation time on a daily 24 h basis. For Model 3, the predicted daily cough frequency was compared with the daily observation count.

## 3. Results

### 3.1. Behavioral Classification Results

The classification results for the calf feeding-related behaviors using Model 1 are shown in Table 2. This model demonstrated excellent overall performance, with a TPR rate of 93.49%, a TNR of 97.54%, a PPV of 93.70%, and an F1 score of 93.59%. Specifically, Model 1 performed best when recognizing natural suckling behavior, achieving an F1 score of 96.88%, with high TPR (96.75%), TNR (99.50%), and PPV (97.01%). Furthermore, the F1 score for classifying rumination behavior reached 95.07%, while the F1 scores for feeding behavior and other behaviors were 91.89% and 90.51%, respectively. Except for other behaviors, Model 1’s PPV was slightly higher than its TPR.

Model 2 focused on determining the postural states of the calves, and its results are shown in Table 3. This model exhibited outstanding overall performance, with an F1 score as high as 97.78%. In particular, the F1 score for the lying posture was 98.45%, and the F1 score for the standing posture was 97.11%, indicating that both postures could be recognized effectively.

Model 3 concentrated on detecting coughing behavior in the calves, and the results are presented in Table 4. Although Model 3’s performance was slightly weaker compared to the previous two models, its overall performance remained good, with a TPR and TNR of 85.49%, a PPV of 85.61%, and an F1 score of 85.55%. The F1 score for the coughing class was 78.61%, which was slightly lower than the F1 scores of the other two models. However, its TNR reached 92.57%, demonstrating that this model excelled at excluding non-coughing instances.

**Figure 5:**
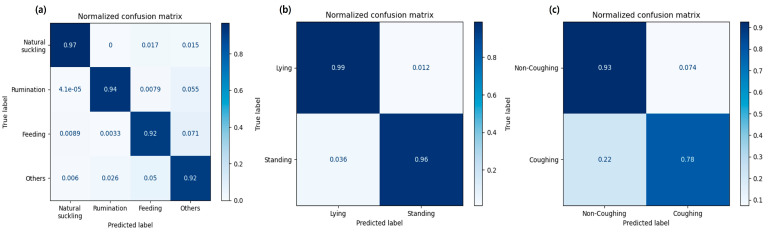
Normalized confusion matrices for three behavior classification models: (**a**) Model 1, classifying natural suckling, rumination, feeding, and others; (**b**) Model 2, classifying lying and standing; and (**c**) Model 3, classifying non-coughing and coughing. Results were evaluated on the test set.

The positive and negative classifications of each behavior class in each model were visualized using confusion matrices, as shown in Figure 5.

### 3.2. ROC Curves with AUC Results

**Figure 6:**
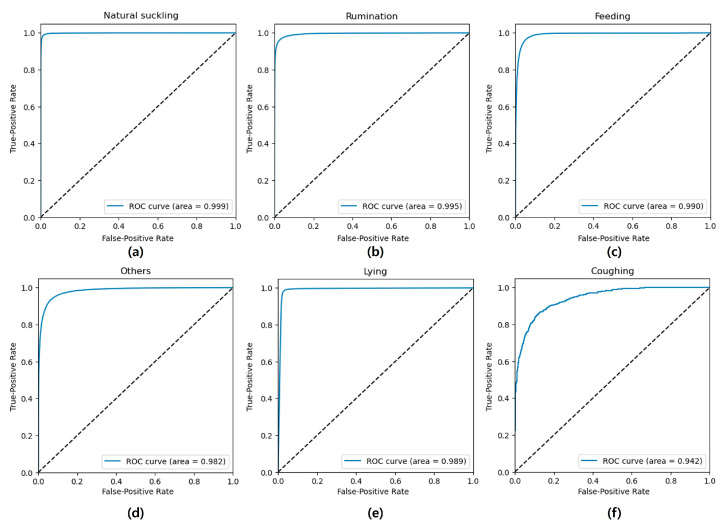
ROC curves with AUC for three behavior classification models: Model 1, classifying (**a**) natural suckling, (**b**) rumination, (**c**) feeding, and (**d**) others; Model 2, classifying (**e**) lying (also for standing); and Model 3, classifying (**f**) coughing. Results were evaluated on the test set.

The ROC curves and AUC for each category are shown in Figure 6. In Model 1, the overall performance was high for each category. Natural suckling behavior exhibited the best performance, with an AUC of 0.999, followed by rumination (AUC of 0.995), feeding (AUC of 0.990), and other behaviors (AUC of 0.982). In Model 2, due to the complementary nature of the lying and standing states, their ROC curves and AUC values were found to be identical, with an AUC of 0.989. Similarly, Model 3 demonstrated a good performance in classifying coughing behavior, with an AUC of 0.942.

### 3.3. Correlation Coefficient Results

**Figure 7:**
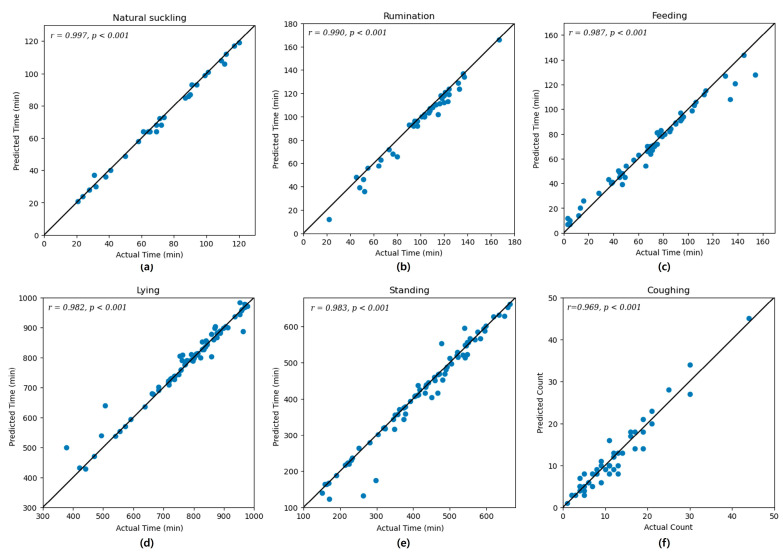
Linear relationships between predicted values and actual values for three behavior classification models: Model 1, showing (**a**) natural suckling, (**b**) rumination, (**c**) feeding; and Model 2, showing (**d**) lying and (**e**) standing. The graphs display the relationship between actual time and predicted time. For Model 3, which classifies (**f**) coughing, the graph shows the relationship between actual count and predicted count. Results were evaluated on the test set.

To validate the models, Pearson correlation coefficients were calculated for each class, and the results are shown in Figure 7. In Model 1, natural suckling exhibited the highest correlation (r = 0.997), followed by rumination (r = 0.990) and feeding (r = 0.987). In Model 2, the correlations for lying and standing were r = 0.982 and r = 0.983, respectively. In Model 3, the correlation for coughing was r = 0.969.

## 4. Discussion

This study aimed to address the critical challenge of simultaneously monitoring multiple behaviors in pre-weaned beef calves within the CCC system. We developed and validated a technique for monitoring multiple behaviors based on machine learning algorithms and a collar-mounted sensor. Traditionally, the simultaneous monitoring of multiple behaviors has necessitated the deployment of several sensors, potentially imposing a burden on the animals [12]. The placement of sensors directly impacts both the accuracy of behavior recognition and the practical applicability of the monitoring system [44]. In this context, collar-mounted devices have emerged as an ideal choice due to their ease of deployment and minimal interference with the animals’ daily activities [45]. On the other hand, as the number and diversity of behaviors to be recognized increase, the identification and classification performances of models tend to decline [46]. To overcome this limitation, our study employed three independent yet complementary machine learning models. This approach enabled more effective handling of complex behavioral patterns and potential behavioral overlaps, thereby enhancing overall recognition accuracy and reliability.

In Model 1 of the current study, we focused on key feeding-related behaviors of calves, including natural suckling, feeding, and rumination. The accurate recognition of natural suckling behavior is particularly crucial for assessing the production, health, and welfare of both cows and calves in the CCC system. Although recent studies have attempted to utilize sensor technology to identify suckling behavior in small ruminants, limitations persist. Carslake et al. [34] successfully employed accelerometers to detect non-nutritive (accuracy: 94.96%) and nutritive suckling (accuracy: 96.44%) in dairy calves within an ACFS. Despite this, the effectiveness of detecting natural suckling behavior within the CCC system remains unclear, as the suckling patterns in CCC systems differ substantially from ACFS. In ACFS, calves typically suckle from artificial teats at fixed positions, while in CCC systems, calves can naturally suckle from their mothers in various positions and angles, making behavior classification more challenging. Kour et al. [16] noted that collar-mounted sensors struggle to effectively differentiate natural suckling from other calf behaviors and proposed a shift to halter-mounted sensors. However, the practicability of halter-mounted sensors and their potential impact on animal welfare warrant further evaluation. In contrast, Kuźnicka et al. [47] successfully identified suckling behavior in lambs using collar-mounted sensors, but their focus was limited to a single behavior, and they did not achieve synchronous monitoring of multiple behaviors. More recently, Price et al. [45] endeavored to simultaneously monitor various behaviors in lambs, including suckling, using harness-mounted sensors. Nevertheless, there was room for improvement in the performance metrics, such as the F1 score (80.38%). Building upon these previous findings, our study aimed to address the limitations of sensor placement, monitoring multiple behaviors, and recognition performance. Using collar-mounted sensors, our approach achieved a high performance in recognizing natural suckling behavior in calves, with an accuracy of 99.10%, an F1 score of 96.88%, an AUC-ROC curve of 0.999, and an r value of 0.997. Despite having the lowest proportion of data among the classes in the model (15.30%), clear features were identified to distinguish natural suckling behavior. This finding represents substantial progress in monitoring natural suckling behavior in calves using collar-mounted sensors and indicates the potential for application in other young ruminants, such as lambs, goats, and fawns.

Choosing the appropriate weaning time is essential for optimizing the growth and rumen development of calves and the reproductive performance of cows [48]. This requires farm managers to not only understand the natural suckling behavior of calves but also to grasp their feeding and rumination behaviors [22,46]. Recent research on using accelerometer sensors to detect feeding and rumination behaviors in calves has primarily focused on halter-mounted or ear-mounted positions. The halter-mounted position has been shown to have advantages in identifying biting or chewing motions in calves because it is better for capturing mandibular movements [49]. However, the reliability of ear-mounted sensors for detecting feeding and rumination behaviors in calves remains unclear. Reynolds et al. [50] suggested that one commercial ear-mounted sensor could measure feeding time in heifers with reasonable accuracy (r = 0.88) but advised caution when using its rumination time function (r = 0.63). Conversely, another commercial ear-mounted sensor demonstrated the opposite pattern (a rumination F1 score of 83.6% and a feeding F1 score of 64.7%) [51]. Interestingly, two studies using sheep indicated that collar-mounted sensors were superior to ear-mounted and mouth-mounted positions when classifying grazing and rumination behaviors [52,53]. To our knowledge, no studies have investigated the use of collar-mounted sensors to detect feeding and rumination behaviors in calves. In the present study, an accuracy of 95.76%, an F1 score of 91.89%, an AUC-ROC curve of 0.990, and an r value of 0.987 were achieved for feeding behavior; for rumination behavior, an accuracy of 97.36%, an F1 score of 95.07%, an AUC-ROC curve of 0.995, and an r value of 0.990 were achieved. These superior results in recognizing natural suckling, feeding, and rumination behaviors may be attributed to the integration of gyroscopes in addition to the accelerometers. Previous studies have shown that gyroscopes provide superior classification performance when identifying dynamic behaviors [54]. In addition, for other behaviors, an accuracy of 93.79%, an F1 score of 90.51%, and an AUC-ROC curve of 0.982 were also achieved. These findings suggest that future work could further subdivide other dynamic behaviors, such as playing and grooming.

In Model 2, we primarily distinguished between two states of calves: lying and standing. The reason for separating Models 1 and 2 was that the dynamic behaviors in Model 1 may occur simultaneously with the behaviors in Model 2. For example, calves can perform natural suckling while standing and can also ruminate while lying down. The results show that Model 2 exhibited excellent classification performance in recognizing both the lying and standing states (accuracy values of 97.98% for both; F1 scores of 98.45% and 97.11%, respectively; an AUC-ROC curve of 0.989 for lying; and r values of 0.982 and 0.983, respectively). With Model 3, we focused on the coughing behavior of calves. BRD is a major challenge affecting the health and growth of calves, and early detection is crucial for timely and targeted treatment [55]. Previous studies have primarily monitored calves using accelerometers to detect indirect signs of BRD, such as reduced feeding behavior or decreased lying bouts [56,57]. However, direct detection of the clinical symptom of coughing was carried out by monitoring coughing sounds using microphones [58,59]. In this study, we achieved a reasonable recognition of coughing behavior, with an accuracy of 88.88%, an AUC-ROC curve of 0.942, and an r value of 0.969. However, the TPR (78.41%), the PPV (78.82%), and the resulting F1 score (78.61%) for the coughing class were moderate. In this experiment, the proportion of samples with coughing (26.05%) was significantly lower than that of samples without coughing (73.95%), indicating a class imbalance issue. To address this, we applied oversampling techniques during training. However, the observed F1 score difference between the coughing (78.61%) and non-coughing (92.49%) classes suggests that the imbalance still may have impacted the model’s learning process [60]. For future research, we plan to obtain more actual coughing data to balance the classes or adjust the class weights during model training to further enhance coughing detection performance. Furthermore, while the F1 score for coughing (78.61%) was reasonable, the high TNR (92.57%) indicated a strong performance in accurately identifying non-coughing instances. Thus, it is expected that misclassification of non-respiratory situations could be substantially reduced, thereby improving the accurate identification of individuals with BRD. It is worth noting that in addition to BRD, diseases in calves such as NCD are also often accompanied by changes in patterns of behavior and activity [61]. This indicates a need to leverage existing and future sensor data to incorporate more behavioral and activity level indicators and further develop decision models for disease detection.

The methodology developed in this study contributes to the field in several aspects. Primarily, it has the potential to offer farm managers a means for monitoring the daily behavioral patterns of calves, which could contribute to the refinement of production management strategies. Furthermore, the capability to detect abnormal behaviors, such as coughing, may serve as an indicator for the early identification of diseases, including BRD. Additionally, it shows promise not only in potentially facilitating animal welfare assessment but also in supporting continuous monitoring following animal welfare certification. Future research directions could further enhance this approach. Potential avenues include expanding the range of recognizable behaviors while maintaining high accuracy and addressing the potential for concept drift in different environmental contexts.

## 5. Conclusions

In conclusion, this study developed and validated a machine learning-based technique for simultaneous monitoring of multiple behaviors in pre-weaned beef calves using collar-mounted sensors integrating a six-axis IMU (accelerometers and gyroscopes). The three complementary models demonstrated high performance in classifying feeding-related behaviors, postural states, and coughing events. This technology has the potential to contribute to the optimization of calf management strategies and facilitate animal welfare assessment in CCC systems.

## Figures and Tables

**Table 1:** Descriptions of each type of collected behavior.

Behavior	Description
Natural suckling	The continuous action of a calf trying to suckle its mother’s milk.
Feeding	The calf approaches the feed trough, lowers its head, chews the feed, and swallows.
Rumination	The sequential action of regurgitating a feed bolus from the rumen, chewing it repeatedly, and then swallowing.
Lying	A condition in which the legs, knees, lower abdomen, or thighs, excluding the bottoms of the hooves, are in sufficient contact with the ground.
Standing	A condition in which the bottoms of the hooves touch the ground and fully support the calf’s body weight.
Coughing	The sudden and forceful expulsion of air in response to mucus or foreign materials in the airways or lungs. The sides of the stomach momentarily swell and contract, and at the same time, the face extends forward and then retracts. The tongue may be momentarily exposed outside the mouth while coughing.

**Table 2:** Classification performance for Model 1 in recognizing calf feeding-related behaviors.

Behavior Class	Accuracy (%)	TPR (%)	TNR (%)	PPV (%)	F1 Score (%)
Natural suckling	99.10	96.75	99.50	97.01	96.88
Rumination	97.36	93.74	98.71	96.43	95.07
Feeding	95.76	91.68	97.20	92.10	91.89
Others	93.79	91.80	94.74	89.26	90.51
Overall	96.50	93.49	97.54	93.70	93.59

Results were evaluated on the test set.

**Table 3:** Classification performance for Model 2 in recognizing calf postural states.

Behavior Class	Accuracy (%)	TPR (%)	TNR (%)	PPV (%)	F1 Score (%)
Lying	97.98	98.82	96.43	98.08	98.45
Standing	97.98	96.43	98.82	97.80	97.11
Overall	97.98	97.63	97.63	97.94	97.78

Results were evaluated on the test set.

**Table 4:** Classification performance for Model 3 in recognizing calf coughing behavior.

Behavior Class	Accuracy (%)	TPR (%)	TNR (%)	PPV (%)	F1 Score (%)
Non-coughing	88.88	92.57	78.41	92.41	92.49
Coughing	88.88	78.41	92.57	78.82	78.61
Overall	88.88	85.49	85.49	85.61	85.55

Results were evaluated on the test set.

## Data Availability

The original contributions of this study are incorporated within the article. For further information or requests regarding the data, please contact the corresponding author.

## Supplementary Materials

The following supporting information can be downloaded at: https://www.mdpi.com/article/10.3390/ani14223278/s1, Figure S1: Average monthly highest and lowest temperatures for Farms A and B during the experiment period; Table S1: Nutrient composition of starter feeds and hays in two experimental farms.
