# Supplementary material for: Monitoring Multiple Behaviors in Beef Calves Raised in Cow–Calf Contact Systems Using a Machine Learning Approach
Source: Animals (Basel). 2024 Nov 14;14(22):3278. doi: 10.3390/ani14223278 (PMC11590895; doi:10.3390/ani14223278)
Supplement: Supplementary file 1 [file animals-14-03278-s001.zip › animals-3265633-supplementary.pdf]

Supplementary Materials:

Table S1. Nutrient composition of starter feeds and hays in two experimental farms.

Nutrient composition	Milk replacer*	Starter feed**	Concentrate***	Timothy hay****	Oat hay
DM (%)	96.0	90.1	87.9	90.1	90.4
Crude protein (% of DM)	24.0	24.5	17.0	14.8	5.4
Crude fiber (% of DM)	-	15.0	8.0	33.8	33.6
Acid detergent fiber (% of DM)	-	-	11.0	37.7	40.2
Neutral detergent fiber (% of DM)	-	-	22.0	67.1	63.4
Total fat (% of DM)	20.0	5.0	3.7	1.1	1.2
Calcium (% of DM)	0.7	0.8	0.9	0.2	0.2
Phosphorous (% of DM)	0.6	0.8	0.5	0.1	0.2
Total digestible nutrients (% of DM)	-	-	73.0	-	-

*The milk replacer is used for calves on farm B, while the calves on farm A are fed their mother's milk.**Both farms used the same commercial feed as the starter (Special baby, Farmsco Inc, South Korea). ***Concentration is used for weaned calves on farm B (Impact young calf, Farmsco Inc, South Korea). ****The hay in the farm A is oat while the farm B is timothy.

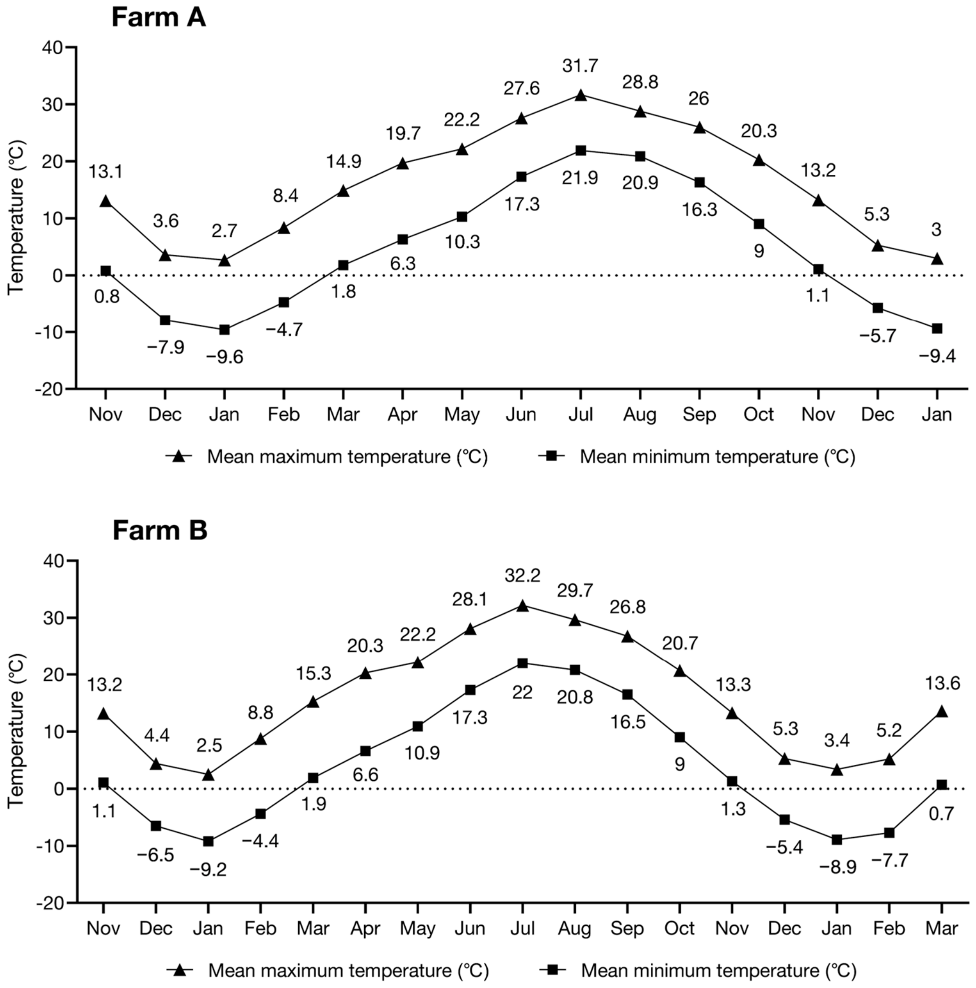

Figure S1. Average monthly highest and lowest temperatures for Farms A and B during the experiment period.
